# Successful treatment of chemotherapy-induced linear bullous lichen planus with dupilumab

**DOI:** 10.1016/j.jdcr.2026.05.005

**Published:** 2026-05-11

**Authors:** Aalia Syed, Hon Fung Alvin Leung, Brandon Tan, Wei Melbourne, Dedee F. Murrell

**Affiliations:** a Faculty of Medicine, University of New South Wales, Sydney, NSW, Australia; b Department of Dermatology, St. George Hospital, Sydney, NSW, Australia; c Bristol Medical School, University of Bristol, Bristol, UK

**Keywords:** bullous lichen planus, corticosteroids, docetaxel, dupilumab, treatment

## Introduction

Bullous lichen planus (BLP), a rare variant of lichen planus (LP), is characterized by tense, pruritic vesicles and bullae arising on or proximal to preexisting LP lesions, most frequently located in the oral mucosa or lower limbs.^1^ The etiology of BLP is unclear, although it is thought to arise from immune-mediated CD4+ T cell activation and CD8+ T cell-driven keratinocyte apoptosis.^1^ BLP is distinct from lichen planus pemphigoides (LPP), an autoimmune blistering disease, and may be distinguished both clinically and through diagnostic tests (Table I).

**Table I:** Distinguishing between BLP and LPP^1^

Feature	Disease	
	BLP	LPP
Pathogenesis	Cell-mediated (CD4+ T cell activation and CD8+ T cell-driven keratinocyte apoptosis), resulting in secondary subepidermal blistering.	Autoantibody production against BP180 and/or BP230.
Clinical features	Vesiculobullous lesions secondarily arise on or close to existing LP plaques, often in a localized distribution.	Papulosquamous and vesiculobullous lesions classically arise on unaffected skin or occasionally on existing LP lesions, often in a generalized distribution.
Histopathology	Classical LP features: hyperkeratosis, saw-tooth acanthosis, lymphocytic infiltrate in the DEJ, apoptotic or dyskeratotic keratinocytes (Civatte or colloid bodies) and interface dermatitis.	Subepidermal blistering and eosinophilic infiltrate with or without histological evidence of LP.
Immunofluorescence	Usually negative or shows nonspecific IgM deposition at the DEJ.	IgG and C3 deposition at the DEJ.
Serology	Seronegative.	Circulating BP180 and/or BP230 antibodies.

*BLP*, Bullous lichen planus; *DEJ*, dermal-epidermal junction; *LP*, lichen planus; *LPP*, lichen planus pemphigoides.

## Case report

A 57-year-old man of Greek heritage presented in May 2020 with an 18-month history of a blistering rash over the lower limbs, manifesting as tense, pruritic, edematous bullae that frequently ruptured causing painful erosions and crusting. The rash was suspected to be secondary to docetaxel chemotherapy for metastatic prostate cancer as there was a temporal relationship between the initiation of docetaxel and the onset of the patient’s cutaneous symptoms. Notably, there was further progression of the rash with ongoing docetaxel therapy, which was administered over 6 cycles from November 2018 to March 2019 due to prioritization of his cancer management. During this time he was treated with topical betamethasone ointment, psoralen and ultraviolet A (PUVA) therapy, and hexachlorophene antibacterial wash, although this led to minimal disease improvement.

Past medical history was significant for metastatic prostate adenocarcinoma with perineural invasion and bone metastases affecting 11 vertebrae (cT3b N0 M1b, Gleason score 4 + 5 = 9), for which he received external beam radiotherapy to the prostate and spine, docetaxel chemotherapy and hormone therapy. His medications included leuprorelin injection 6-monthly and oxycodone 80 mg twice daily. Physical examination revealed violaceous, polymorphous bullae in a serpentine configuration along the anterior and posterior shins bilaterally. There were no oral mucosal lesions. Bullous Pemphigoid Disease Area Index (BPDAI) score was 20/240 for activity (A) and 2/120 for damage (D).

**Fig 1:**
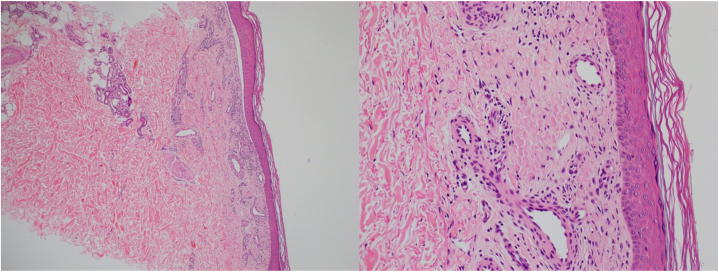
H&E stained section demonstrating parakeratosis, epidermal atrophy, Civatte bodies in the basal epidermis, mild interface inflammation with scarring, subepidermal vesicles and lymphocytic infiltrate in the papillary dermis.

Three punch biopsies were taken; 2 from the right lower leg for routine histopathology and direct immunofluorescence, and 1 additional biopsy from the left lower leg for routine histopathology. Histopathology revealed parakeratosis, epidermal atrophy, Civatte bodies in the basal epidermis, mild interface inflammation with scarring, subepidermal vesicles and lymphocytic infiltrate in the papillary dermis (Fig 1). There was no eosinophilic infiltrate. Direct immunofluorescence was negative for IgG, IgA, IgM, albumin, fibrinogen C3 and C1q.

Serologic testing, performed on 3 separate occasions over 2 years, for BP180, BP230, DSG1, DSG3, envoplakin, and collagen VII autoantibodies were consistently negative. These findings confirmed a diagnosis of linear BLP and excluded a diagnosis of LPP. A timeline illustrating the initiation of docetaxel, symptom development and diagnosis is demonstrated in Table II.

**Table II:** Timeline of bullous lichen planus (BLP) presentation and diagnosis

Date	Event
November 2018	Docetaxel chemotherapy for metastatic prostate cancer initiated.
November 2018	Onset of tense, pruritic, edematous, bullous skin lesions.
March 2019	Docetaxel ceased after 6 cycles.
May 2020	Presented to our dermatology clinic.
May 2020	Three punch biopsies from the lower limbs were performed for routine histopathology and direct immunofluorescence, revealing findings consistent with bullous lichen planus (BLP).
June 2022, December 2022 & January 2023	Serology was negative for BP180, BP230, DSG1, DSG3, envoplakin, and collagen VII autoantibodies.

The patient was initially commenced on narrowband ultraviolet B (UVB) therapy 3 times weekly for 8 weeks, topical clobetasol propionate under occlusion and acitretin 10 mg twice daily. However, at review in August 2020, he reported poor tolerance to occlusive topical treatment due to increased sweating and pruritus. UVB therapy had also resulted in new active linear vesicles overlying areas of previous scarring for unclear reasons. He was commenced on prednisolone 25 mg daily in July 2021, increasing to 50 mg daily when pruritus was uncontrolled, alongside triamcinolone injections and acitretin until May 2022, which provided partial flattening of the lesions. Acitretin was subsequently switched to isotretinoin 20 mg daily due to minimal clinical improvement.

Unfortunately by mid-2022, he developed steroid-induced type 2 diabetes mellitus, osteoporosis and Cushing syndrome due to prolonged administration of systemic corticosteroids, prompting tapering of prednisone to 15 mg daily. Concurrently, metformin 1000 mg twice daily, semaglutide 1.5 mg weekly and denosumab injection 6-monthly were commenced. Due to poor disease control and persistent pruritus, he received rituximab 1000 mg intravenously on 2 occasions in June and July 2022; however, therapy was discontinued due to deranged liver function tests.

Given the refractory course of his BLP, dupilumab 300 mg subcutaneous injection fortnightly was initiated in March 2023 alongside continued isotretinoin and prednisone. His clinical photographs and BPDAI activity, damage and pruritus scores are demonstrated in Table II. The patient gradually improved with no further blistering and reduced pruritus. By August 2023, the lesions had begun to transition from erythematous bullae to dull plaques (Table III). Whilst on dupilumab, prednisone was gradually weaned from 15 mg daily to 10 mg daily over 10 m. At his most recent review in September 2025, examination revealed no active bullae or vesicles, smaller and less painful erosions, and significant post-inflammatory hyperpigmentation (BPDAI A1 D2).

**Table III:** Clinical photographs demonstrating the progression of lower limb BLP over time on dupilumab, as assessed by BPDAI total activity, damage and pruritus scores

Month (Year)	Clinical photographs of the (a) anterior lower limb and (b) posterior lower limb demonstrating BLP	BPDAI total activity and damage scores	BPDAI pruritus component (VAS)
March (2023): first dose of dupilumab		Activity: 20 Damage: 2	23/30
August (2023): 4 mo of dupilumab		Activity: 6 Damage: 2	16/30
September (2025): most recent review		Activity: 1 Damage: 2	21/30∗

*BLP*, Bullous lichen planus; *BPDAI*, Bullous Pemphigoid Disease Area Index; *VAS*, visual analogue scale.

∗ Patient admitted to missing 2 doses of dupilumab prior to this visit, resulting in a greater than normal BPDAI pruritus score. Patient verified that no other doses of dupilumab were missed, and that his pruritus is under control after resuming dupilumab.

## Discussion

There are currently no defined treatment regimens for BLP. As BLP is often thought to be a severe form of LP involving extensive inflammation,^2^ potent topical corticosteroids are typically administered as first-line therapy.^3^ There have also been sporadic reports on the use of systemic corticosteroids such as betamethasone oral mini-pulse therapy, mycophenolate mofetil, dapsone and acitretin in BLP.^3^ Notably, there exists 1 case report documenting successful treatment of BLP using adalimumab.^4^

In our patient, conventional BLP treatments including phototherapy, topical and systemic corticosteroids as well as acitretin provided little to no therapeutic benefit. Apart from disease flares, our patient also experienced multiple complications from prolonged systemic corticosteroid use and is not suitable for further therapy with rituximab. Drawing from limited evidence on the treatment of similar conditions, he was started on dupilumab as an attempt to control his symptoms and to limit eruptive episodes.

Dupilumab, an inhibitor of interleukin 4 (IL-4) and 13 (IL-13) signaling, has been increasingly utilized to treat many dermatological conditions due to its regulatory effects on type 2 inflammation. There is growing evidence supporting its role as an effective treatment for bullous pemphigoid (BP),^5^ and a recent systematic review has explored its therapeutic value in LP, particularly in cases that were drug-induced or recalcitrant, though there were also reports of dupilumab-induced LP conversely.^6^ Anecdotal evidence from case reports also support its use in treating both bullous^7^ and non-bullous^8^ variants of LPP. In both BP and LP, it has been suggested that dupilumab’s action on IL-4 and IL-13 signaling can downregulate the pathway of Th2 cells, which is a type of CD4+ cell that plays a role in the inflammatory processes of these 2 diseases.^5^^,^^9^ As a rare variant of LP, BLP shares similarities with its more prevalent counterpart on pathophysiological fronts, with the disease processes of both diseases involving CD4+ T-cell (Th1 and Th2) mediation, evidenced by inflammatory T-cell infiltrates on histology.^1^^,^^9^ Thus, it can be theorized that there may be an overlap between the influences of dupilumab on BP, LP and BLP given its antagonistic effect on Th2 cells, potentially explaining the regression of skin lesions on our patient upon administration of dupilumab.

In conclusion, our case demonstrates that dupilumab may have a role in the management of BLP due to its action on Th2 cells and subsequent downregulation of cytokines IL-4 and IL-13. Further studies are warranted to ascertain the relationship between dupilumab and BLP.

## Conflicts of interest

None disclosed.
